# Binding Sites for Acylated Trehalose Analogs of Glycolipid Ligands on an Extended Carbohydrate Recognition Domain of the Macrophage Receptor Mincle*[Fn FN2]

**DOI:** 10.1074/jbc.M116.749515

**Published:** 2016-08-19

**Authors:** Hadar Feinberg, Neela D. S. Rambaruth, Sabine A. F. Jégouzo, Kristian M. Jacobsen, Rasmus Djurhuus, Thomas B. Poulsen, William I. Weis, Maureen E. Taylor, Kurt Drickamer

**Affiliations:** From the ‡ Departments of Structural Biology and Molecular and Cellular Physiology, Stanford University School of Medicine, Stanford, California 94305,; the § Department of Life Sciences, Imperial College, London SW7 2AZ, United Kingdom, and; ¶ Chemical Biology Laboratory, Department of Chemistry, Aarhus University, DK-8000 Aarhus, Denmark

**Keywords:** carbohydrate-binding protein, glycolipid, lectin, mycobacteria, tuberculosis

## Abstract

The macrophage receptor mincle binds to trehalose dimycolate on the surface of *Mycobacterium tuberculosis*. Signaling initiated by this interaction leads to cytokine production, which underlies the ability of mycobacteria to evade the immune system and also to function as adjuvants. In previous work the mechanism for binding of the sugar headgroup of trehalose dimycolate to mincle has been elucidated, but the basis for enhanced binding to glycolipid ligands, in which hydrophobic substituents are attached to the 6-hydroxyl groups, has been the subject of speculation. In the work reported here, the interaction of trehalose derivatives with bovine mincle has been probed with a series of synthetic mimics of trehalose dimycolate in binding assays, in structural studies by x-ray crystallography, and by site-directed mutagenesis. Binding studies reveal that, rather than reflecting specific structural preference, the apparent affinity of mincle for ligands with hydrophobic substituents correlates with their overall size. Structural and mutagenesis analysis provides evidence for interaction of the hydrophobic substituents with multiple different portions of the surface of mincle and confirms the presence of three Ca^2+^-binding sites. The structure of an extended portion of the extracellular domain of mincle, beyond the minimal C-type carbohydrate recognition domain, also constrains the way the binding domains may interact on the surface of macrophages.

## Introduction

Mincle, the macrophage-inducible C-type lectin, was initially identified as an orphan receptor expressed in macrophages when they are stimulated with bacterial lipopolysaccharide (1). It was subsequently identified as a receptor for trehalose dimycolate, an unusual glycolipid found on the surface of *Mycobacterium tuberculosis* (2, 3). Trehalose dimycolate, also known as cord factor, plays a critical role in the interaction of mycobacteria with macrophages by facilitating establishment of granulomas that allows this pathogen to exist in a latent state in a protected environment within macrophages (4). Consequences of trehalose dimycolate binding to mincle include production and secretion of interleukin 6 and tumor necrosis factor α (2). Mincle is a simple type II transmembrane protein with a short cytoplasmic tail that lacks obvious signaling motifs (1). However, it associates with the common Fc receptor γ subunit, which binds to and activates Syk kinase through immunotyrosine activation motifs (5, 6). Downstream signaling events, involving the adaptor molecules CARD9 and Bcl10 and the MALT1 paracaspase, are required for cytokine secretion. The signaling functions of mincle are of interest both for understanding the interaction of mycobacteria with macrophages and to explain the ability of trehalose dimycolate to act as an adjuvant in stimulating the immune response (3).

The extracellular domain of mincle contains a C-type carbohydrate recognition domain (CRD).^3^ Previous structural studies have revealed that the CRD has many of the features seen in other modules of this type, including a primary sugar-binding site centered on a conserved Ca^2+^ that binds to one of the two glucose residues that are linked α1,1 in trehalose (7). The second glucose residue makes additional contact with an extended binding site in the CRD. These structural studies combined with mutagenesis data provide a basis for understanding the interaction of cow mincle with its mycobacterial ligand. Further studies have provided evidence that the active forms of the CRDs in human and mouse mincle are probably similar to that observed in crystals of the cow protein (8, 9). Although a published structure of the human protein shows a somewhat different conformation, this structure lacks the key conserved Ca^2+^ involved in binding sugars (10). Despite the insights that have been obtained into cow, human, and mouse mincle structures, several aspects of the structure of the CRD and the way it interacts with ligands could not be deduced from the previous analysis. The CRD studied previously was truncated at its N terminus. In addition, a secondary Ca^2+^-binding site was occupied by Na^+^, and only the trehalose headgroup of the glycolipid ligand was present in the crystals.

Further studies on the ligand interactions with the extracellular domain of cow mincle reported here define the presence of three Ca^2+^-binding sites and demonstrate several potential ways in which acyl groups attached to trehalose can interact with hydrophobic binding surfaces on the protein. The structure of an extended CRD also provides insight into the portion of the module that is linked to the cell membrane, where it interacts with its signaling partner.

## Results

### 

#### 

##### Effect of 6-OH Substituents on Binding to Mincle

In the mycobacterial glycolipid trehalose dimycolate, the 6-OH groups of both glucose residues of trehalose are esterified to β-branched fatty acids. Two classes of smaller, water-soluble compounds that mimic the ability of trehalose dimycolate to bind to mincle have been investigated. In one class of ligands, the long mycolic acid chains have been replaced by shorter, linear fatty acids (7, 10). These compounds are synthetically accessible using a lipase under non-aqueous conditions. Because these ligands are water-soluble, it is possible to measure affinities for mincle by employing a competition assay in which binding of labeled mannose-conjugated serum albumin is displaced by the competing ligands under conditions in which the inhibition constants, *K_I_*, approximate the dissociation constants for the competing ligands (11). The addition of acyl chains of increasing length enhances the affinity of these compounds relative to unmodified trehalose. Other structurally distinct ligands are based on the natural product brartemicin in which modified forms of benzoic acid are esterified to the glucose residues (12).

**FIGURE 1.**
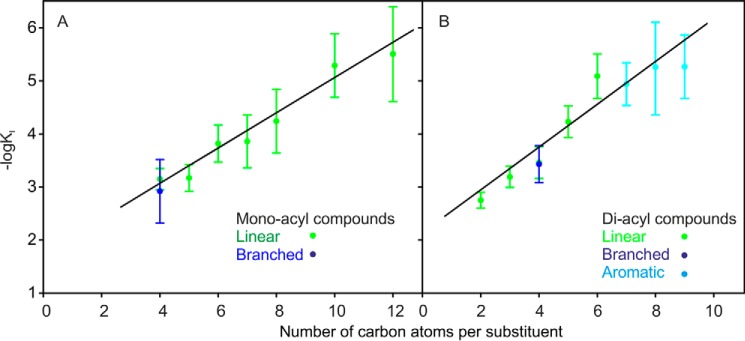
**Relationship between apparent affinity for mincle and total size of acyl groups attached to 6-OH groups of trehalose.** Affinities for monoacylated (*A*) and diacylated (*B*) trehalose derivatives were determined in binding competition assays. Binding of radiolabeled mannose-conjugated serum albumin to biotin-tagged CRD from mincle immobilized in streptavidin-coated wells was detected in the presence of soluble competing ligands. *K_I_* values measured in the competition experiments closely approximate the *K_D_* values (11). Straight lines were fitted to the data by least-squares fitting. Results are reported as the means ± S.D. for *n* = 3–4 separate experiments, each performed in duplicate.

Despite the variation in the structures of the acylated groups, the general pattern that emerges from these studies is that binding is enhanced by increasing the size of the groups attached to trehalose. To investigate this relationship in more detail, the range of the linear acyl groups tested with bovine mincle was extended to include diacylated ligands bearing 5- and 6-carbon linear fatty acids and monoacylated ligands with 7-, 8-, 10-, and 12-carbon linear fatty acids. An additional class of compounds in which a β-branched fatty acid was attached to trehalose using the lipase approach was also generated. The *K_I_* values for the novel compounds are provided in Table 1, and the estimated values for the association constants for all of the ligands that have been tested with bovine mincle are summarized in Fig. 1. These graphs reveal a nearly linear relationship between the logarithm of the *K_I_* values and the number of carbon atoms in the acyl substituents, which appears to hold regardless of the arrangement of the substituents as linear, branched, or in aromatic rings. Because the negative of the logarithm of the *K_I_* would increase linearly with free energy of binding, the increase in apparent affinity corresponds to a linear increase in binding energy as a function of number of carbon atoms in the substituents.

**TABLE 1:** **Inhibition of ^125^I-Man-BSA binding to mincle by acylated derivatives of trehalose** Results are reported as means ± S.D. for *n* = 3–4 separate experiments, each performed in duplicate.

Compound	*K_I_*	*K_I_*_,trehalose_/*K_I_*_, compound_
	μ*m*	
Trehalose	2200 ± 300	
Longer diacylated derivatives		
Divalerate	59 ± 2	34 ± 4
Dihexanoate	8.2 ± 0.3	250 ± 30
Longer monoacylated derivatives		
Monoheptanoate	137 ± 18	22 ± 2
Monooctanoate	58 ± 8	52 ± 5
Monodecanoate	5.1 ± 0.6	310 ± 30
Monododecanoate	3.1 ± 0.5	530 ± 100
Branched chain derivatives		
Monoisobutyrate	1100 ± 100	2.5 ± 0.2
Diisobutyrate	370 ± 40	7.3 ± 0.8

To ensure that affinities for monomeric ligands were measured, the ability of the compounds with longer acyl chains to form micelles was investigated by dynamic light scattering. No micelle formation for any of the diacylated compounds was detected at concentrations 10-fold higher than those employed in the binding assays. Critical micelle concentrations of 3 mm and 0.2 mm were estimated for the 10- and 12-carbon monoacylated derivatives. These concentrations are >10-fold higher than the highest concentrations employed in the assays and are >100-fold higher than the *K_I_* values. Thus, the observed enhancement in affinities for the compounds with longer acyl chains could not be ascribed to multivalent binding to micelles.

When plotted as a function of the number of carbon atoms attached to each glucose residue, the line for diacylated trehalose ligands has a slope ∼20% higher than the line for monoacylated derivatives. This minimal increase in the slope for the diacyl compounds, indicating that the second substituent does not contribute very much to affinity, would suggest that there may be only limited interaction of the second substituent with the CRD. In fact, if ligands with a single acyl chain bind in a single preferred orientation, in which the acyl substituent can interact favorably with the CRD, then the diacylated version could bind in either orientation, which could provide some enhancement in the affinity without any interaction of the second substituent with the CRD.

A key point that emerges is that the size dependence of the affinities is broadly consistent regardless of the structural details of the substituents. It is also interesting that the affinity increase continues up through 12-carbon linear fatty acids, whereas the previous modeling suggested that an 8-carbon fatty acid would be sufficient to extend from the trehalose to the edge of the CRD. Thus, although it is possible that the observed increases in affinity reflect increasing interactions between the surface of the CRD and the acyl substituents on the trehalose, decreasing solubility of the larger derivatives may also contribute to the apparent increase in affinity.

##### Structure of Mincle Complexed with a Monoacylated Trehalose Derivative

**FIGURE 2.**
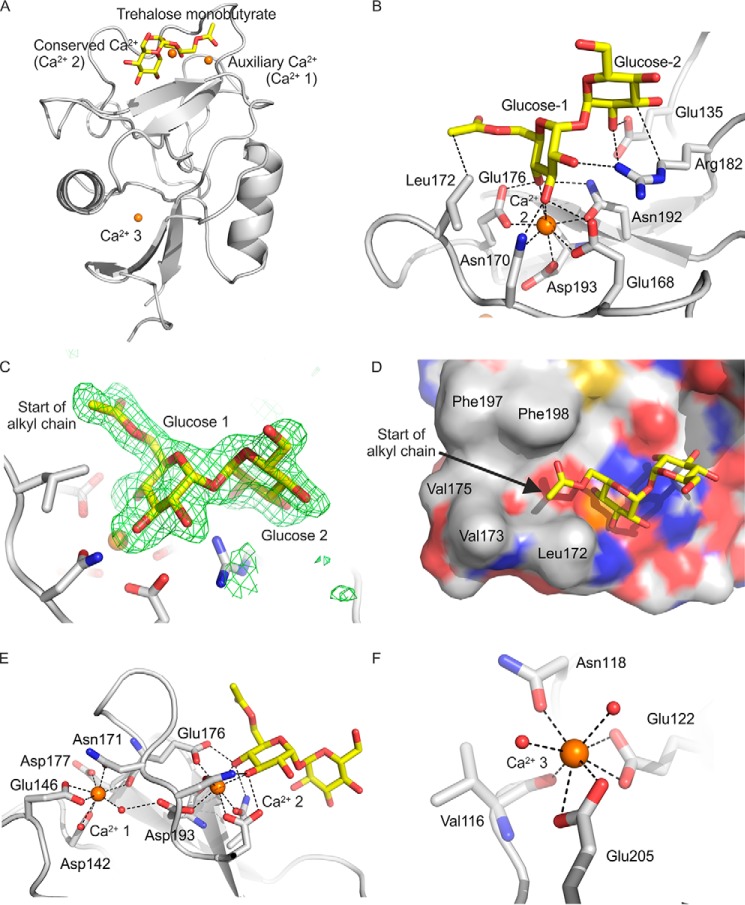
**Structure of mincle CRD bound to trehalose monobutyrate.** *A*, overall structure of complex. *B*, close-up view of ligand-binding site, with glucose residue 1 in the primary sugar-binding site, ligated to the conserved Ca^2+^ (Ca^2+^ 2), and glucose residue 2 in the secondary sugar-binding site. *C*, Fo-Fc electron density omit map, calculated by omitting the trehalose monobutyrate ligand from the model, contoured at 3.0 σ, showing partial monobutyrate structure attached to the 6-OH group of glucose residue 1. *D*, surface of CRD showing the position of the carboxyl end of the monobutyrate substituent at the end of the hydrophobic groove formed by residues Phe-197 and Phe-198 on one side and Leu-172 and Val-173 on the other. *E*, close-up view showing amino acid residues ligated to the auxiliary Ca^2+^ (Ca^2+^ 1), including residues that bridge to the conserved Ca^2+^ (Ca^2+^ 2). *F*, close-up view of Ca^2+^ 3, showing amino acid side chain and backbone ligands. Copy C of the protein is shown. Protein in schematic representations as well as carbon atoms of stick and surface representations are presented in *gray*, carbon atoms of the sugars are *yellow*, oxygen atoms are *red*, nitrogen atoms are *blue*, and Ca^2+^ ions are *orange*. The electron density map is represented as *green mesh*.

Co-crystals of trehalose monobutyrate and the CRD of mincle were examined to explore the arrangement of simple, linear acyl substituents attached to the 6-OH of glucose residues in trehalose (Tables 2 and 3). The electron density for the trehalose portion of bound trehalose monobutyrate is well defined in all three crystallographically independent copies (designated A, B, and C), and as shown in Fig. 2, *A* and *B*, it matches exactly that observed for unmodified trehalose; that is, glucose residue 1 occupies the primary sugar-binding site typical of C-type lectins, liganded to the conserved Ca^2+^ (Ca^2+^ 2), and glucose residue 2 makes additional contacts with an adjacent secondary sugar-binding site.

**TABLE 2:** **Crystallographic data statistics**

Data	Minimal CRD from mincle with monobutanoyl trehalose	Extended CRD from mincle with trehalose	Extended CRD from mincle with brartemicin	Extended CRD from mincle with brartemicin analog
Space group	P2_1_	P3_1_21	P3_1_21	P3_1_21
Unit cell parameters	*a* = 66.5 Å *b* = 46.7 Å	*a* = *b* = 97.8 Å	*a* = *b* = 98.3 Å	*a* = *b* = 98.4 Å
	*c* = 77.9 Å ß = 101.2°	*c* = 45.2 Å	*c* = 45.6 Å	*c* = 45.7 Å
Number of copies in asymmetric unit	3	1	1	1
Beamline for data collection*^a^*	SSRL 11-1	APS 23ID-B	SSRL 12-2	SSRL 12-2
Wavelength (Å)	1.03317	1.03320	0.97946	0.97946
Resolution Å (last shell)	38.2-2.10 (2.21-2.10)	48.9-2.60 (2.72-2.60)	33.5-2.20 (2.27-2.20)	33.5-1.80 (1.83-1.80)
R_sym_ (last shell)*^b^*	8.5 (46.7)	10.2 (48.1)	7.8 (35.7)	5.6 (53.5)
Mean (*I*) half-set correlation (CC_1/2_)	0.998 (0.917)	0.994 (0.759)	0.997 (0.933)	0.998 (0.845)
Mean ((*I*)/σ(*I*)) (last shell)	13.7 (3.5)	9.7 (2.3)	10.8 (4.1)	14.5 (2.9)
% Complete (last shell)	98.0 (95.1)	98.6 (97.4)	99.5 (97.8)	99.5 (94.2)
Number of unique reflections	27266	7737	13146	23855
Average multiplicity (last shell)	4.5 (4.3)	4.3 (4.0)	5.0 (5.0)	5.0 (4.8)

*^a^* SSRL, Stanford Synchrotron Radiation Lightsource; APS, Advanced Photon Source.

*^b^* R_sym_ = Σ_h_Σ_i_(|I_i_(h) − 〈I(h)〉|)/Σ_h_Σ_i_ I_i_(h), where I_i_(h) = observed intensity, and 〈I(h)〉 = mean intensity obtained from multiple measurements.

**TABLE 3:** **Crystallographic refinement statistics** The atomic coordinates and structure factors (codes 4ZRV for CRD with trehalose monobutyrate; 4ZRW for extended CRD with trehalose; 5KTH for extended CRD with brartemicin; 5KTI for extended CRD with brartemicin analog) have been deposited in the Protein Data Bank.

Data	Minimal CRD from mincle with monobutanoyl trehalose	Extended CRD from mincle with trehalose	Extended CRD from mincle with brartemicin	Extended CRD from mincle with brartemicin analog
Number of reflections				
Working set	25887	7375	12264	22251
Test set	1367	358	624	1116
R_free_*^a^*	20.8	20.8	21.0	20.4
R_work_*^a^*	16.3	15.2	16.8	17.8
Average B factor (Å^2^)	16.9	43.2	40.8	31.5
Bond length rmsd (Å)	0.014	0.015	0.014	0.015
Angle rmsd (°)	1.11	1.16	0.98	0.91
Ramachandran plot (% in each region*^b^* preferred/allowed/outliers)	90.6/9.1/0.3	94.4/5.6/0.0	95.6/4.4/0.0	94.9/5.1/0.0

*^a^* R_work_ and R_free_ = Σ |F_o_ − F_c_|/ΣF_o_, where F_o_ = observed structure factor amplitude, and F_c_ = calculated structure factor amplitude for the working and test sets, respectively.

*^b^* As defined in Coot.

Extra density elongated off O6 of glucose residue 1 was also observed for copies B and C. For copy C, the carbonyl group and one more carbon from the butyrate moiety were added to the model, but the remainder of the butyrate was not evident in the electron density map (Fig. 2*C*). The ω torsion angle for the C5-C6 bond in glucose residue 1 is changed to 62° from the value of 190° that is seen in the previously published structure of underivatized trehalose bound to mincle (7). Thus, there is a shift between the two common staggered conformations that avoid steric clashes (13). The change in ω angle and the presence of the carbonyl group at 100% occupancy suggests that the glucose residue with the attached butanoyl group is preferentially bound in the primary sugar-binding site. As suggested in previous modeling studies, the point of attachment of the acyl chain places it near the hydrophobic groove formed by residues Phe-197 and Phe-198 on one side and Leu-172 and Val-173 on the other side (Fig. 2*D*), which would be consistent with interaction of the hydrocarbon portion of the acyl chain with the hydrophobic groove. However, despite the fact that the monobutyrate ligand has an affinity for mincle that is roughly 2.8-fold higher than trehalose (7), no additional density was observed for the remainder of the acyl chain. The absence of electron density suggests that this portion of the ligand may not have a fixed conformation.

Crystals of the monobutyrate complex of the CRD from mincle were obtained at pH 5.6, whereas the complex with trehalose studied previously was obtained at pH 5.0 (7). The increase in pH appears to have resulted in full occupancy of three Ca^2+^ sites (Fig. 2, *E* and *F*). Ca^2+^ 2 is generally conserved in C-type CRDs, as they bind sugars by coordination of hydroxyl groups to this Ca^2+^. Ca^2+^ 1 is referred to as an auxiliary Ca^2+^, as this Ca^2+^ needs to be bound to position side chains of amino acids that ligate Ca^2+^ 2 (Fig. 2*E*). However, in the previously described structure of the trehalose-CRD complex, Ca^2+^ 1 is replaced by Na^+^ (7). A similar situation, in which high concentrations of Na^+^ compared with Ca^2+^ results in a Na^+^ replacing one of the Ca^2+^ bound to a C-type CRD, has been previously observed in the structure of surfactant protein A (14). This observation plus the presence of Ca^2+^ at near physiological concentrations suggests that the structure with Ca^2+^ at site 1 represents the physiologically relevant conformation around the sugar-binding site.

The auxiliary Ca^2+^ (Ca^2+^ 1) is observed in numerous other C-type CRDs, including those in mannose-binding protein, pulmonary surfactant proteins A and D, the asialoglycoprotein receptor, DC-SIGN, DC-SIGNR, and the scavenger receptor C-type lectin (14–19). The presence of both the conserved and auxiliary Ca^2+^ is required to form fully functional sugar-binding sites in these proteins. However, only the asialoglycoprotein receptor, the scavenger receptor C-type lectin, and dendritic cell immunoreceptor have a Ca^2+^ that corresponds to Ca^2+^ 3 in mincle (Fig. 2*F*) (17, 19, 20). A role for this third Ca^2+^, which is distant from the sugar-binding site (Fig. 2*A*), remains to be established.

##### Organization of an Extended CRD of Mincle

**FIGURE 3.**
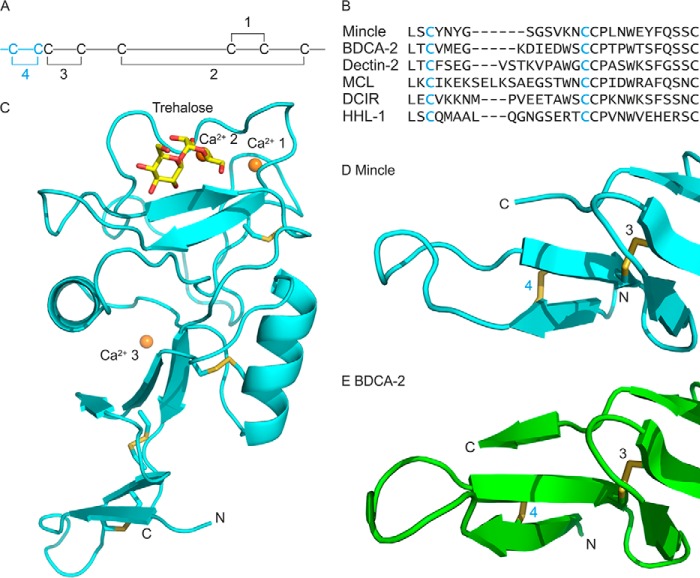
**Structure of the extended CRD from mincle.** *A*, arrangement of disulfide bonds in C-type CRDs. The additional fourth disulfide bond in the extended CRD is highlighted in *cyan. B*, sequence comparisons of N-terminal extensions in CRDs. Sequences shown are from bovine mincle and human blood dendritic cell antigen 2, human dectin-2, macrophage C-type lectin (*MCL*, also designated dectin-3), human dendritic cell immunoreceptor (*DCIR*), and human hepatic lectin-1 (*HHL-1*, major subunit of the asialoglycoprotein receptor). *C*, overall structure of the extended CRD, showing bound trehalose and three Ca^2+^. Structures of the N-terminal extensions of mincle (*D*) and BDCA-2, PDB entry 4ZES (*E*) are shown. Proteins in schematic representations are shown in *cyan* for the extended CRD from mincle and *green* for BDCA-2. Disulfide bonds are highlighted in *yellow*, and other atoms are colored as in Fig. 2.

Further structural analysis of the CRD from mincle in complex with ligands was undertaken using an extended CRD that encompasses an additional disulfide bond at the N terminus compared with the previously analyzed minimal CRD. Mincle is one of a subset of C-type CRDs that contains eight cysteine residues linked in four disulfide bonds (Fig. 3, *A* and *B*). The two C-terminal nested disulfide bonds 1 and 2 are present in all the sugar-binding C-type CRDs, and disulfide 3 is present in many CRDs. However, the additional N-terminal disulfide 4 is found only in the closely related asialoglycoprotein receptor and macrophage galactose receptor, in the dendritic cell immunoreceptor, and in the group of receptors that associate with the FcRγ chain: mincle, blood dendritic cell antigen 2 (BDCA-2), dectin-2, and macrophage C-type lectin (21). As with the minimal CRD, the extended CRD used in these studies contains a threonine residue at position 174 (single nucleotide polymorphism rs135158086) rather than isoleucine, as found in the reference sequence (NCBI accession number XP_002687869).

In crystals of the extended CRD in complex with trehalose (Fig. 3*C*), the core CRD structure observed in previous analysis of the truncated CRD is preserved (7). All three Ca^2+^ sites are also fully occupied in the extended CRD, which was crystallized at pH 8.5. The main structural feature of the N-terminal extension is a pair of β strands connected by a loop. One of the β strands interacts with the C-terminal residues of the CRD. Interaction of the C-terminal sequence with an N-terminal β strand is a common feature of C-type CRDs, but the extension adds an adjacent β strand and elongates the CRD because of the presence of the loop. Pairing of the N-terminal β strands is stabilized by the presence of disulfide bond 4 between cysteine residues in the two strands.

Until recently no structural information was available for extended CRDs, but a structure for human BDCA-2 has been recently described (22). Although there is almost no similarity in the sequences of the residues that lie between the cysteine residues that form disulfide bond 4 in mincle and BDCA-2, the arrangement of the extension is similar (Fig. 3, *D* and *E*). Much of the sequence divergence is in the loop between the paired β strands. Comparison of the sequences of the other CRDs that contain disulfide 4 (Fig. 3*B*) suggests that the size and conformation of this loop is likely to vary significantly.

##### Structures of Mincle Complexed with Diacylated Trehalose Derivatives

**FIGURE 4.**
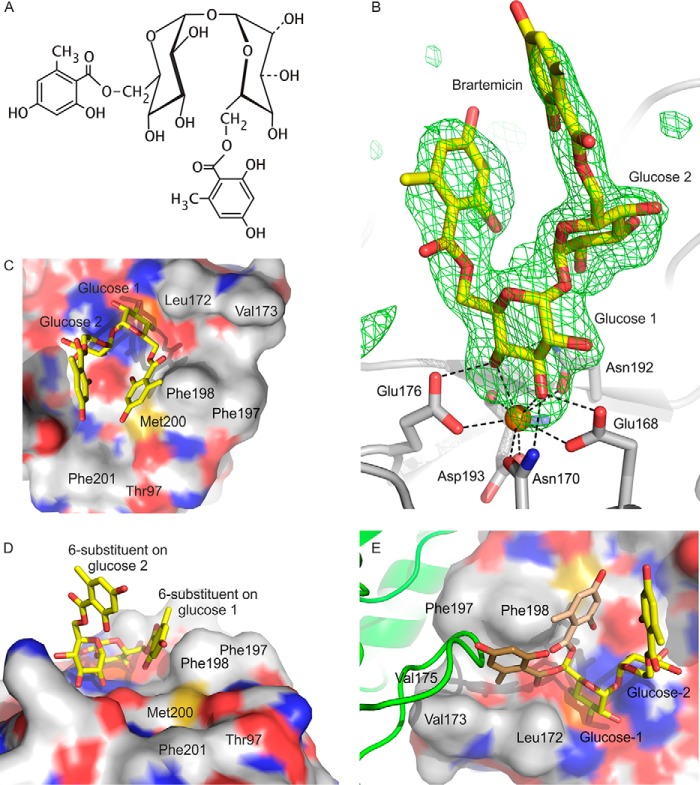
**Structure of brartemicin bound to the extended CRD of mincle.** *A*, chemical structure of brartemicin. *B*, omit map showing the position of brartemicin bound to the extended CRD from mincle. Electron density in the Fo-Fc map is contoured at 3.0 σ. *C* and *D*, surface representation of brartemicin-binding site from above and side. *E*, view of the binding site showing a loop from a symmetry-related molecule (*green*) occupying part of the hydrophobic groove adjacent to glucose residue 1. The arrangement of the 6-substituent attached to glucose residue 1 observed in the crystal structure is shown in *light brown*, and a model for an alternative conformation that occupies the same space as the loop from the adjacent molecule in the crystal structure is shown in *dark brown*. Atoms are represented as in Fig. 2.

Trehalose-containing ligands for mincle that are based on the natural product brartemicin were also investigated. The relatively rigid structures of the aromatic rings in brartemicin (Fig. 4*A*) suggested that it might be a better crystallization target than the compounds with flexible acyl substituents. The structures of the extended CRD bound to either natural brartemicin or a synthetic analog showed that the position of the trehalose portion of the ligand corresponds to that seen in the previous structures of trehalose and trehalose monobutyrate with the minimal CRD and of trehalose with the extended CRD (Fig. 4*B*). However, in the brartemicin structure, the ω torsion angle of the C5-C6 bond in glucose residue 1, in the primary sugar-binding site, is 207°, similar to the conformation observed for free trehalose in the binding site (7).

Electron density corresponding to both of the aromatic rings in brartemicin was observed. The better-defined ring, which is attached to the 6 position of glucose residue 1, is packed against the side chain of Phe-198, which along with Phe-197 forms a knob projecting from the surface of the CRD and constitutes one side of the hydrophobic groove into which an acyl chain might fit (Fig. 4*C*). However, as a result of the altered ω torsion angle, the brartemicin ring is located on the opposite side of the phenylalanine knob from the relatively narrow hydrophobic groove formed by Phe-197 and Phe-198 on one side and Leu-172 and Val-173 on the other (Fig. 4, *C* and *D*).

The position of the substituents on the benzene ring cannot be unambiguously determined from the experimental data, as the ring could be rotated 180° compared with the orientation shown in Fig. 4*D* so that the hydroxyl and methyl groups that flank the carboxyl group would be interchanged. In the orientation shown the hydroxyl group interacts with the surface created by the edge of Phe-198, Glu-135, and Met-200, which lie below. The methyl group would be exposed to the solvent. However, the methyl group could also be accommodated under the ring, with the hydroxyl group exposed to the solvent. Either way, the ability to accommodate a hydroxyl group on the carbon atom that is 1 atom removed from the carbonyl group is interesting, as this portion of brartemicin mimics the disposition of groups in trehalose dimycolate (12).

The structure of the mincle-brartemicin complex reveals that hydrophobic substituents on the 6-OH group of glucose residue 1 can be positioned outside the hydrophobic groove. Several factors may contribute to this arrangement. First, the observed structure of brartemicin may reflect an inherent tendency of the ligand to flex so that the two aromatic substituents come close to each other. In addition, in the crystal structure the hydrophobic groove is partially occupied by a loop that spans residues 152–154 of a neighboring molecule in the crystal lattice (Fig. 4*E*). Blocking of the hydrophobic groove in this crystal form may in part account for the fact that a clear conformation of the brartemicin substituent is observed, whereas the substituent is not observed for trehalose monobutyrate in the other crystal form; if multiple conformations are possible, blocking of one conformation in the hydrophobic groove would favor the remaining conformations. In the case of brartemicin, rigidity of the ligand would result in a single preferred conformation in this crystal form. Reduction in affinity for brartemicin resulting from blocking of one binding conformation would be compensated by favorable interactions forming the crystal lattice.

Electron density is also observed corresponding to part of the second aromatic ring of brartemicin, attached to the 6-OH group of glucose 2 (Fig. 4*B*). The density in this case is weaker, albeit sufficient to establish the orientation of the ring. In contrast to the other substituent on the trehalose, this ring projects away from the CRD, and this portion of the ligand does not make any contacts with the surface of the protein. The weaker electron density of the second substituent in brartemicin indicates that its position is not fully fixed.

**FIGURE 5.**
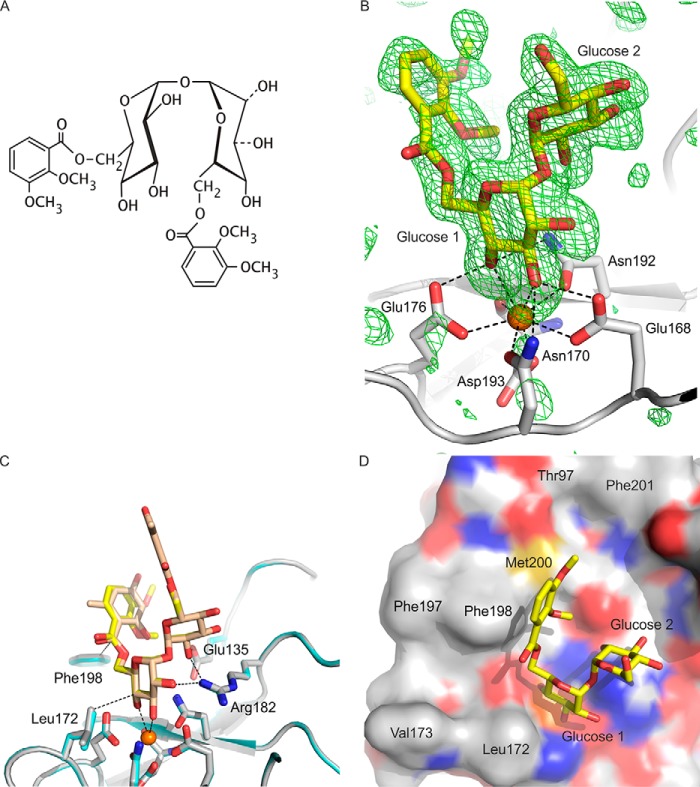
**Structure of brartemicin analog bound to the extended CRD of mincle.** *A*, chemical structure of brartemicin analog. *B*, Fo-Fc omit map showing the brartemicin analog with electron density contoured at 3 σ. *C*, overlay of brartemicin and brartemicin analog structures. In this panel, carbon atoms in brartemicin are colored *light brown*, and carbon atoms of the analog are shown in *yellow*, with protein carbon atoms of the CRD shown in *gray* for the brartemicin complex and *cyan* for the analog complex. *D*, surface representation of brartemicin analog in the binding site of mincle. Atoms are represented as in Fig. 2.

The structure of mincle bound to a synthetic analog of brartemicin was also examined. This compound demonstrates the lack of requirement for specific substituents on the aromatic rings, as these have been modified (Fig. 5*A*) without significantly changing the affinity for mincle (12). In the crystals of the mincle analog complex, the aromatic ring attached to glucose residue 1 is also observed in a similar position to that seen in brartemicin (Fig. 5, *B* and *C*). In the analog structure, the ω torsion angle for the C5-C6 bond in glucose residue 1 is 203°, making it very similar to the orientation seen in brartemicin. In this case, the orientation of the ring can be established unambiguously based on the position of the methoxy group adjacent to the carboxyl group. This group is tucked into the space formed by the aromatic ring of the analog, the side of Phe-198, and the sulfur and adjacent carbons of Met-200 as well as Glu-135 (Fig. 5*D*). The complete absence of electron density corresponding to the second substituent in the complex with the analog is consistent with flexibility in the position of this substituent.

##### Mutagenesis of Secondary Hydrophobic Surface

It was proposed previously that an acyl chain attached to the 6-OH group of glucose residue 1 would lie in the groove formed by Phe-197 and Phe-198 on one side and Leu-172 and Val-173 on the other (7). The position of the visible portion of trehalose monobutyrate bound to the CRD is consistent with this arrangement. However, although the position of the substituent would be relatively constrained in the groove, the absence of defined density for most of the alkyl chain suggested that, instead of taking up one specific orientation, the bound chains might have multiple modes of interaction with the surface of the CRD. The observed interactions of brartemicin with the surface on the other side of the Phe-197–Phe-198 knob, including Met-200 (Fig. 4*C*), raised the possibility that a linear acyl chain could also interact with this region some of the time. This additional nonpolar region is extended by Phe-201 and modeling suggests that an 8-carbon chain might extend as far as the methyl groups on Thr-158 as well.

**FIGURE 6.**
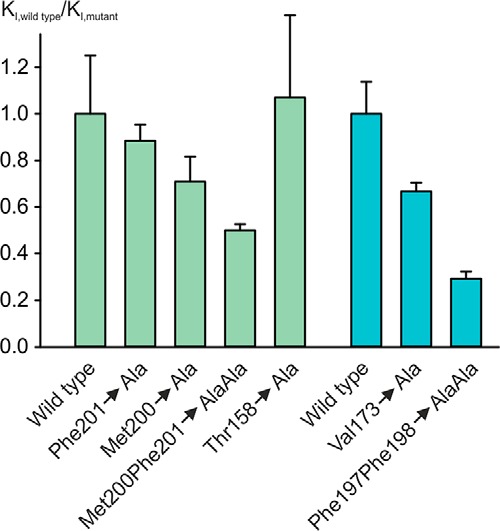
**Mutagenesis of hydrophobic binding surface.** Affinity of mutants for mono-octanoyltrehalose was measured in binding competition assays. *K_I_* values for each mutant are normalized to the *K_I_* value for trehalose with that mutant to eliminate the impact of changes on the affinity for trehalose. The only instance in which there is more than a 20% change in the affinity for trehalose is in the case of changing Met-200 to alanine, which increases the *K_I_* for trehalose by 3.0 ± 0.1-fold. This effect probably reflects the role of Met-200 in positioning Glu-135, which interacts with glucose residue 2 in the extended sugar-binding site. Results in *blue*, recalculated from (7), are for the hydrophobic groove, whereas results in *green* are for the additional hydrophobic surface that interacts with brartemicin. The graph shows the ratio of the normalized *K_I_* values for the wild type CRD compared with the mutants, so smaller numbers reflect reduced affinity. Results are reported as the means ± S.D. for *n* = 3–4 separate experiments, each performed in duplicate.

The contribution of this more-extended surface to binding of trehalose with a linear acyl chain was examined by mutating each of these residues and comparing binding of the resulting mutated proteins to monooctanoyltrehalose and trehalose (Fig. 6). The results show that individual changes have a small effect and that changing both Met-200 and Phe-201 simultaneously results in a 2-fold decrease in the relative affinity for trehalose octanoate. This decrease in affinity compares to a 1.5-fold reduction in affinity resulting from mutation of Val-173 on one side of the hydrophobic groove and a 3.4-fold reduction in affinity resulting from mutation of both Phe-197 and Phe-198 (7). Changes in the latter residues might be expected to affect binding either in the groove or at the secondary hydrophobic surface. Thus, the results of both the mutagenesis and structural analysis are consistent with the suggestion that there is flexibility in the interaction of acyl substituents with the CRD and that both the groove and the additional hydrophobic surface may be involved in binding hydrophobic portions of ligands.

## Discussion

The results reported here confirm that the trehalose headgroup of the acylated structures, which serve as models for the more complex trehalose dimycolate ligand, is anchored in the extended binding site of the mincle CRD, with the two glucose residues occupying identical positions in all of the structures examined. In addition, they provide insights into the dispositions of the acyl groups attached to the 6-OH groups of the two glucose residues. In several of the structures, substituents attached to glucose residue 1 in the primary sugar-binding site are visible, whereas in only one case is density observed for a substituent on glucose residue 2 in the secondary binding site. These results indicate that acyl groups on glucose residue 1 are more likely to contact the surface of the protein than substituents of glucose residue 2.

The binding affinities for mono- and diacylated derivatives as well as preferential binding of the monoacylated derivative in this orientation indicate that interaction of the surface of the protein with the acyl chain on glucose residue 1 provides most of the enhanced affinity of acylated ligands for mincle. Consistent with the interpretation, in the one case in which an acyl substituent attached to glucose 2 is observed, the substituent points away from the surface of the protein. These observations explain the finding that trehalose monomycolate is also a ligand for mincle (23), as the trehalose headgroup would presumably be orientated so that the acyl chain occupies the position near to the protein surface.

A key observation is that acyl groups attached to the 6-OH group of glucose residue 1 can interact with a relatively broad surface on the CRD in mincle and can take on multiple conformations. This interpretation is supported by the absence of discernable electron density for part of the acyl chain in the co-crystals of trehalose monobutyrate, the location of the aromatic rings in the brartemicin and analog structures, and the mutagenesis results. The potential for many different interactions, no one of which is essential for high affinity binding of acylated derivatives of mincle, explains why removal of any one portion of the binding surface results in only modest loss of affinity for these ligands. This interpretation also explains why it is difficult to observe the complete acylated structures in a unique conformation in the crystals.

**FIGURE 7.**
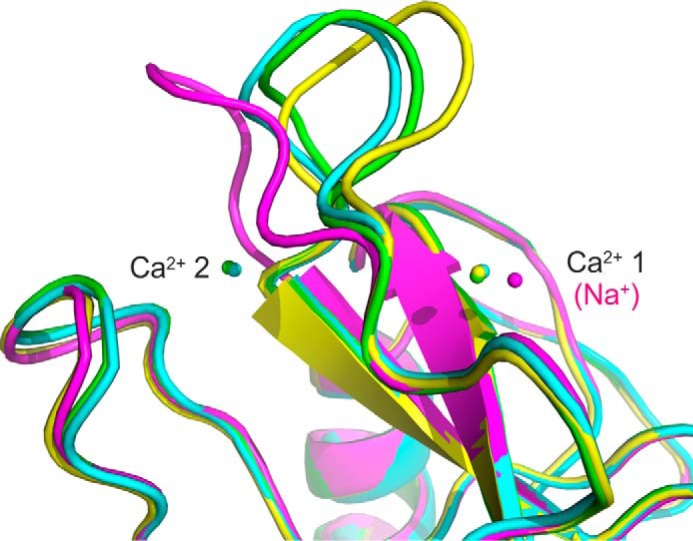
**Flexibility in the side of the hydrophobic groove.** Superposition of mincle CRD structures shows complexes of the minimal CRD with citrate (*magenta*), with trehalose (*yellow*), and with trehalose monobutyrate (*green*) as well as the extended CRD complexed with trehalose (*cyan*).

Comparing the structures of the CRD from mincle in various complexes indicates that part of the CRD, particularly the loop between residues 171 and 177, takes on different conformations under different conditions (Fig. 7). The largest conformational difference was observed in the structure in which the auxiliary Ca^2+^ (Ca^2+^ 1) is missing, which is consistent with previous studies showing that loss of Ca^2+^ ions leads to conformational changes in C-type CRDs (19, 24, 25). However, more subtle differences in the positions of these residues are seen in the presence of the auxiliary Ca^2+^, which suggests that there may be flexibility in the structure that results in changes under different crystallization conditions and in the presence of different ligands. It is interesting to note that residues Leu-172 and Val-173, which form one side of the hydrophobic groove, are located in this loop, so that the types of conformational flexibility observed might influence the precise structure of the groove.

**FIGURE 8.**
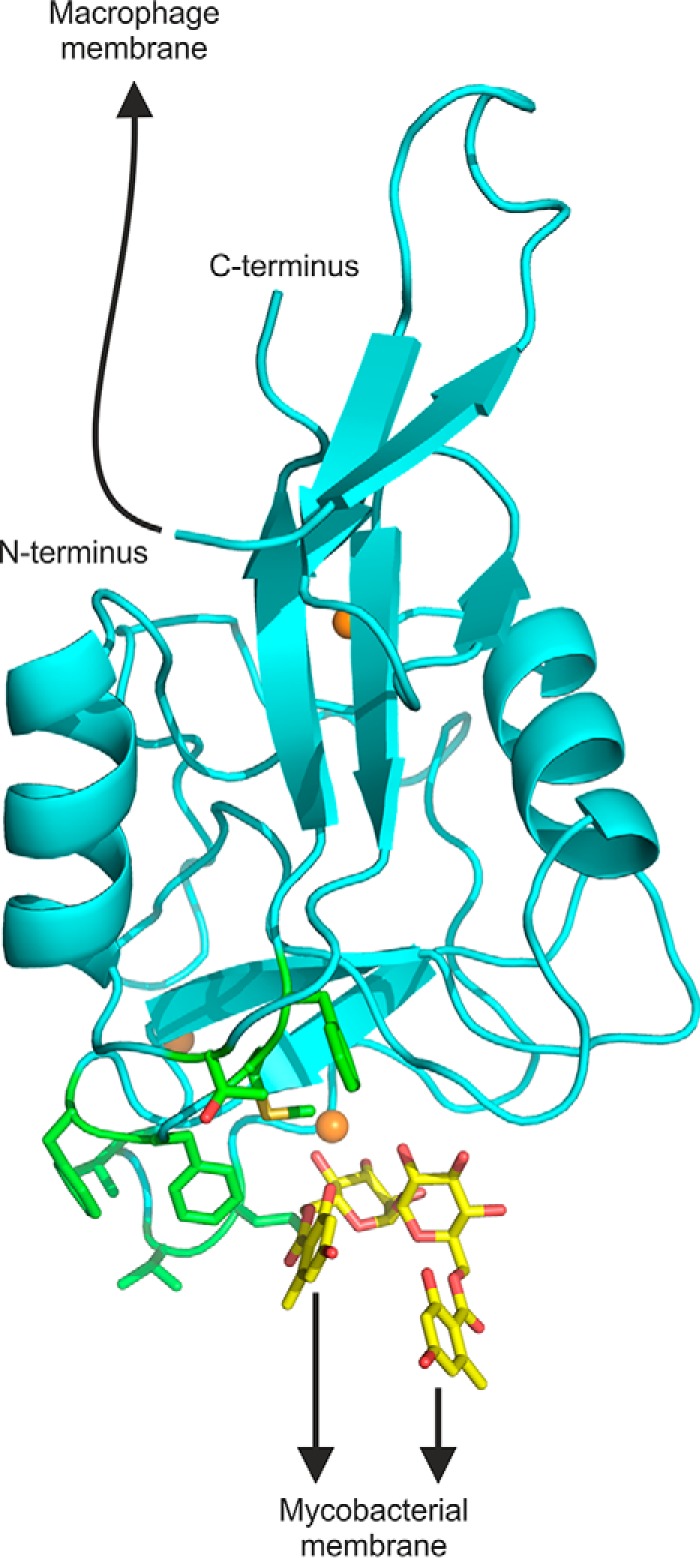
**Model showing orientation of extended N terminus and ligand-binding sites.** A composite model was generated by positioning brartemicin, from the crystal structure of brartemicin with the CRD, in the binding site of the extended CRD with bound trehalose. The model highlights portions of the extended CRD that interact with the macrophage membrane and with glycolipid targets at the surface of mycobacteria. The N terminus is linked to the macrophage membrane by a 19-amino acid sequence not seen in the crystal structure. The binding site for mycobacterial ligands is indicated based on the orientation of brartemicin in the binding site and highlighting of hydrophobic residues implicated in ligand binding in *green*. Other atoms are colored as in Fig. 2.

The CRD in mincle is linked to the remainder of the polypeptide through the N terminus, so the structure of the extended CRD helps to define how sugar-binding sites are orientated at the cell surface for binding to pathogens. If the sugar binding site and the adjacent hydrophobic regions are positioned toward the bacterial membrane, the extended CRD lies along the membrane surface (Fig. 8). In this orientation the N terminus would be connected to the macrophage membrane by the stretch of 19 amino acids, residues 46–64, that lies between the membrane and the CRD. Crystals of the full extracellular domain including this additional portion of the polypeptide did not show any further density beyond that seen in the extended CRD structure (data not shown). Thus, this region may be a flexible linker that facilitates positioning of the CRD to interact with the mycobacterial membrane.

The presence of the N-terminal extension also provides insight into the way that mincle can interact with other polypeptides on the macrophage cell surface. Receptors containing shorter C-type CRDs that lack this extension often form oligomers in which the N-terminal regions interact through a relatively flat surface on this end of the CRD (16, 26, 27). The presence of the extension and the absence of a well defined neck region able to form a typical coiled-coil stalk would prevent the types of interactions seen in these oligomers and may facilitate interactions of the transmembrane domain with common Fc receptor γ subunit rather than between mincle polypeptides.

## Experimental Procedures

### 

#### 

##### Expression of Extended CRD of Mincle

An extended portion of the cDNA for bovine mincle was amplified from a liver cDNA library (United States Biological) using the polymerase chain reaction (Advantage 2 Polymerase Mix, Takara) with forward primer aaggatccgatcttggaggatgattaaatggctgaactctcctgctataatgatggatcagg and backward primer aaagcttcaaatctttctttctggcatttcacaaacccgaaacatattgaagaaac. The resulting fragment was cloned into vector pCR2.1-TOPO (Invitrogen) and sequenced using an Applied Biosystems 310 genetic analyzer. The forward primer includes a BamH1 restriction site and a short peptide linker at the end of the phage T7 gene 10 protein followed by a stop codon, an in-frame methionine codon, and an alanine codon, before residue Glu-64 of bovine mincle. The BamH1 site and an EcoR1 site in the reverse primer were used for cloning into the pT5T expression vector (28). When expressed in *Escherichia coli* strain BL21(DE3), the initiator methionine is removed, leaving an N-terminal alanine residue appended to Glu-64. The extended CRD was expressed as inclusion bodies, renatured, and purified by affinity chromatography on trehalose-Sepharose exactly as for the minimal CRD expressed previously (7).

##### Synthesis of Acylated Trehalose Derivatives

Brartemicin and brartemicin analog were synthesized as previously described (12). Preparation of other acylated forms of trehalose has also previously been documented (7–9), except for four novel compounds. The protocol for synthesis of these compounds followed the general procedure employed before (7), except that the monoacylated forms were favored by reacting 500 mg of trehalose with 0.5 ml of decanoic acid, 0.5 ml of dodecanoic acid, 1 ml of 2-ethyl-butyric acid, or 1 ml of 2-propyl pentanoic acid (Sigma). Monoacylated derivatives were separated by chromatography on 25 ml of silica gel in chloroform/methanol/water (75:25:4), filtered, and assayed as described previously. The four new compounds were characterized by matrix-assisted laser desorption ionization mass spectrometry on an Applied Biosystems 4800 instrument and by one-dimensional proton NMR on a Bruker 400-MHz spectrometer (supplemental Figs. S1 and S2). The ability of mono- and diacylated compounds to form micelles was examined by measuring dynamic light scattering at various dilutions in the buffer used for the binding assays on a Viscotek 802 instrument.

##### Binding Competition Studies

The minimal CRD with a C-terminal biotinylation tag immobilized in streptavidin-coated wells was used for binding competition assays, with ^125^I-mannose-conjugated bovine serum albumin employed as the reporter ligand (7). Each set of assays employed duplicate wells and was repeated three to four times. The reported values are the means ± S.D. for the replicate experiments. Curve fitting was performed with SigmaPlot.

##### Crystallization and Data Collection

Crystals of the minimal cow mincle CRD complexed with trehalose monobutyrate were obtained by hanging-drop vapor diffusion at 22 °C using a mixture of 3 μl of protein solution and 1 μl of reservoir solution. The protein solution consisted of 2.1 mg/ml CRD, 2.5 mm CaCl_2_, and 33 mm trehalose monobutyrate, and the reservoir solution contained 20% polyethylene glycol 4000, 20% 2-propanol, and 0.1 m sodium acetate, pH 5.6. Crystals were frozen directly from the drop in liquid nitrogen for data collection.

Crystals of the extended cow mincle CRD complexed with trehalose were grown by hanging-drop vapor diffusion using a mixture of 0.9 μl of protein solution and 0.9 μl of reservoir solution at 16.5 °C. The protein solution contained 7.2 mg/ml CRD, 5 mm CaCl_2_, 10 mm Tris-Cl, pH 8.0, 25 mm NaCl, and 30 mm trehalose, and the reservoir solution consisted of 2% polyethylene glycol 3350, 0.2 m MgCl_2_, 0.1 m Tris, pH 8.5. Crystals were transferred to a solution containing 30% polyethylene glycol 3350, 0.2 m MgCl_2_, 0.1 m Tris-Cl, pH 8.5, 5 mm CaCl_2_, and 30 mm trehalose before being frozen in liquid nitrogen for data collection. Crystals of the extended CRD with brartemicin were grown from a mixture of 2 μl of protein solution and 2 μl of reservoir solution at 22 °C with 4.1 mg/ml CRD, 5 mm CaCl_2_, 10 mm Tris-Cl, pH 8.0, 25 mm NaCl, and 5 mm brartemicin in the protein solution and 25% polyethylene glycol 3350, 0.2 NaCl, and 0.1 m Bis-tris, pH 5.5, in the reservoir solution. Crystals of the extended CRD with the brartemicin analog were grown from a mixture of 1 μl of protein solution and 1 μl of reservoir solution at 22 °C, with the protein solution comprising 10 mg/ml CRD, 5 mm CaCl_2_, 10 mm Tris-Cl, pH 8.0, 25 mm NaCl, and 5 mm analog, whereas the reservoir solution contained 20% polyethylene glycol 4000, 20% 2-propanol and 0.1 m sodium acetate, pH 5.6. Crystals of the latter two complexes were frozen directly from the drops.

Data from the minimal CRD-trehalose monobutyrate complex were processed with XDS (29) and scaled with SCALA (30). Data for the extended CRD-trehalose complex were processed with MOSFLM (31) and scaled with AIMLESS (32). Data for extended CRD complexed with brartemicin or the brartemicin analog were processed with XDS and scaled with aimless. Statistics are summarized in Table 2.

##### Structure Determination

The first two structures were solved by molecular replacement using the program phaser (33). The model used for molecular replacement was derived from monomer A of Protein Data Bank entry 4KZV, without the saccharide and water molecules. The molecular replacement solution indicated that the space group was P2_1_ with three monomers in the asymmetric unit for the minimal CRD-trehalose monobutyrate complex and space group P3_1_21 with one monomer in the asymmetric unit for the extended CRD-trehalose complex. The maps for both complexes indicated the presence of three Ca^2+^ per monomer: the two Ca^2+^ found in Protein Data Bank entry 4KZV plus a further Ca^2+^site. The presence of the third Ca^2+^ was confirmed by an anomalous difference Fourier map for the higher resolution dataset for the trehalose monobutyrate complex, which revealed three peaks corresponding to the Ca^2+^ in all three copies in the asymmetric unit. The structures for the brartemicin and brartemicin analog complexes were solved by rigid body refinement using the partially refined extended CRD-trehalose structure. The datasets were re-indexed, and R_free_ reflections were chosen based on the reflections chosen for R_free_ in the data for the initial model. Model building and refinement were performed with Coot (34) and PHENIX (35). Refinement included individual positional and isotropic temperature factor refinement. Refinement statistics are shown in Table 3.

##### Mutagenesis of CRD

Mutagenesis was performed by two-step polymerase chain reaction (36) using the extended cDNA clone as template. The mutant proteins were expressed and purified exactly as for the wild type protein.

## Author Contributions

M. E. T. and K. D. designed the study. N. D. S. R., S. A. F. J., and K. D. prepared the trehalose derivatives with alkyl side substituents, expressed and purified the wild type and mutant proteins, and characterized the binding activity. H. F. determined the protein structures. K. M. J., R. D., and T. B. P. prepared brartemicin and the synthetic analog. W. I. W., M. E. T., and K. D. wrote the paper. All authors analyzed the results and approved the final version of the manuscript.

## Supplementary Material

Supplemental Data

## Footnotes

3 The abbreviations used are:

CRD: carbohydrate recognition domain
BDCA-2: blood dendritic cell antigen 2
Bis-tris: 2-[bis(2-hydroxyethyl)amino]-2-(hydroxymethyl)propane-1,3-diol.
