# Supplementary material for: Binding Sites for Acylated Trehalose Analogs of Glycolipid Ligands on an Extended Carbohydrate Recognition Domain of the Macrophage Receptor Mincle
Source: J Biol Chem. 2016 Aug 19;291(40):21222–33. doi: 10.1074/jbc.M116.749515 (PMC5076529; doi:10.1074/jbc.M116.749515)
Supplement: Supplemental Data [file supp_291_40_21222__index.html]

Binding sites for acylated trehalose analogs of glycolipid ligands on an extended carbohydrate-recognition domain of the macrophage receptor mincle — Binding Sites for Acylated Trehalose Analogs of Glycolipid Ligands on an Extended Carbohydrate Recognition Domain of the Macrophage Receptor Mincle — Acylated Trehalose Analogs Binding to Mincle — Supplemental Data 

# Binding Sites for Acylated Trehalose Analogs of Glycolipid Ligands on an Extended Carbohydrate Recognition Domain of the Macrophage Receptor Mincle

## Supplemental Data

- Supplemental data (.pdf, 476 KB) - Supplemental data
