# Supplementary material for: Binding Sites for Acylated Trehalose Analogs of Glycolipid Ligands on an Extended Carbohydrate Recognition Domain of the Macrophage Receptor Mincle
Source: J Biol Chem. 2016 Aug 19;291(40):21222–33. doi: 10.1074/jbc.M116.749515 (PMC5076529; doi:10.1074/jbc.M116.749515)

Supplemental Information

Binding Sites for Acylated Trehalose Analogs of Glycolipid Ligands on an Axtended Carbohydrate-recognition Domain of the Macrophage Receptor Mincle

Hadar Feinberg, Neela D. S. Rambaruth, Sabine A. F. Jégouzo, Kristian M. Jacobsen, Rasmus Djurhuus, Thomas B. Poulsen, William I. Weis, Maureen E. Taylor, and Kurt Drickamer

Figure S1. Mass spectrometry of acylated trehalose derivatives.

Figure S2. Proton NMR spectra of acylated trehalose derivatives.

Figure S1. Mass spectrometry of di-acylated trehalose derivatives. Samples were prepared by spotting together equal volumes of matrix, 2,5-dihydroxybenzoic acid as a 10 mg/ml solution 80% methanol, and sugar derivative, dissolved at approximately 1 mg/ml in water, on a target plate. Mass spectrometry was performed on an Applied Biosystems 4800 matrix assisted-laser desorption time-of-flight mass spectrometer.

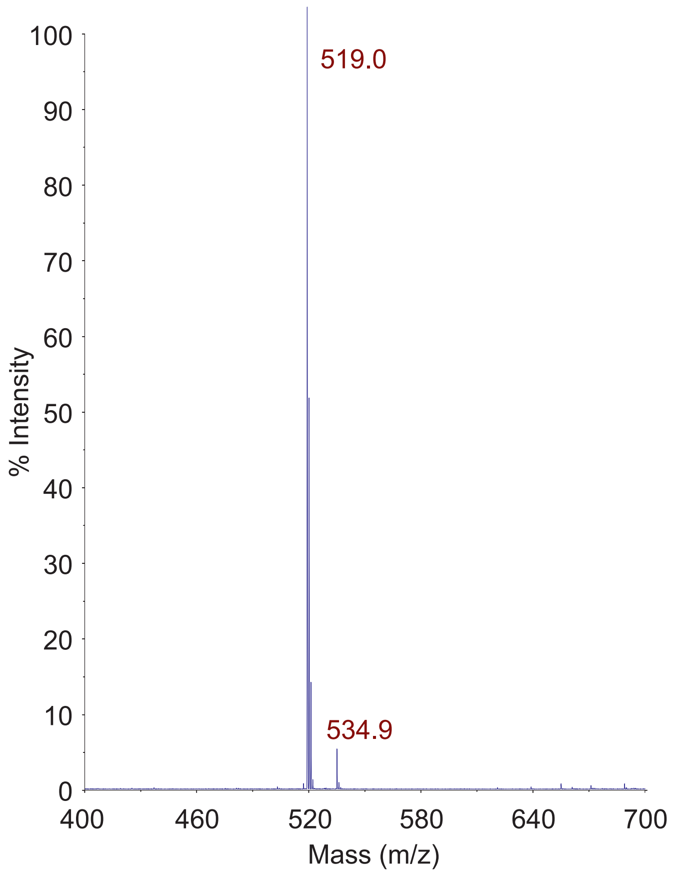

A Trehalose mono decanoate 519(Na) / 535 (K)

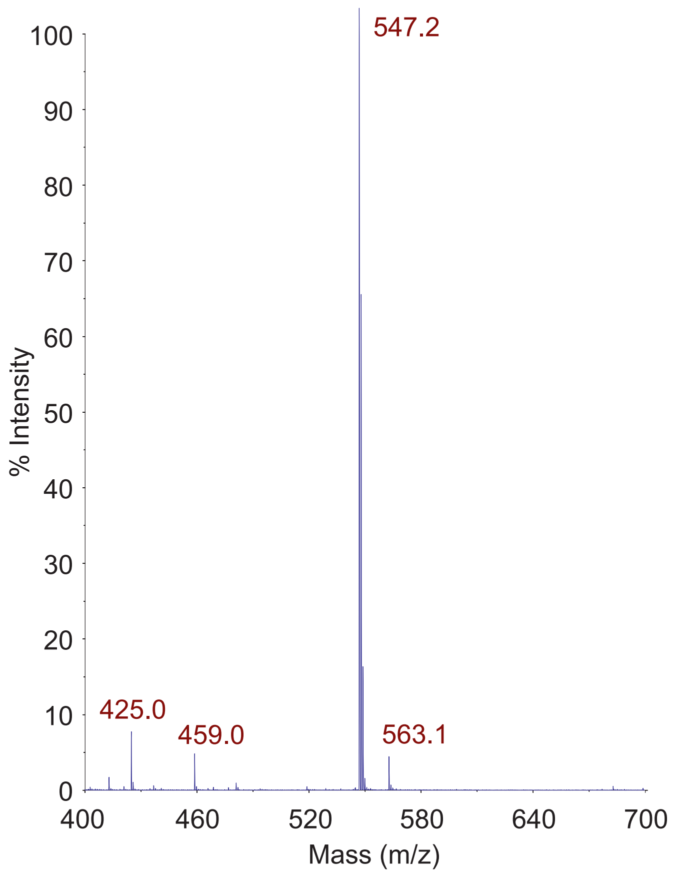

B Trehalose mono dodecanoate 547 (Na) / 563 (K)

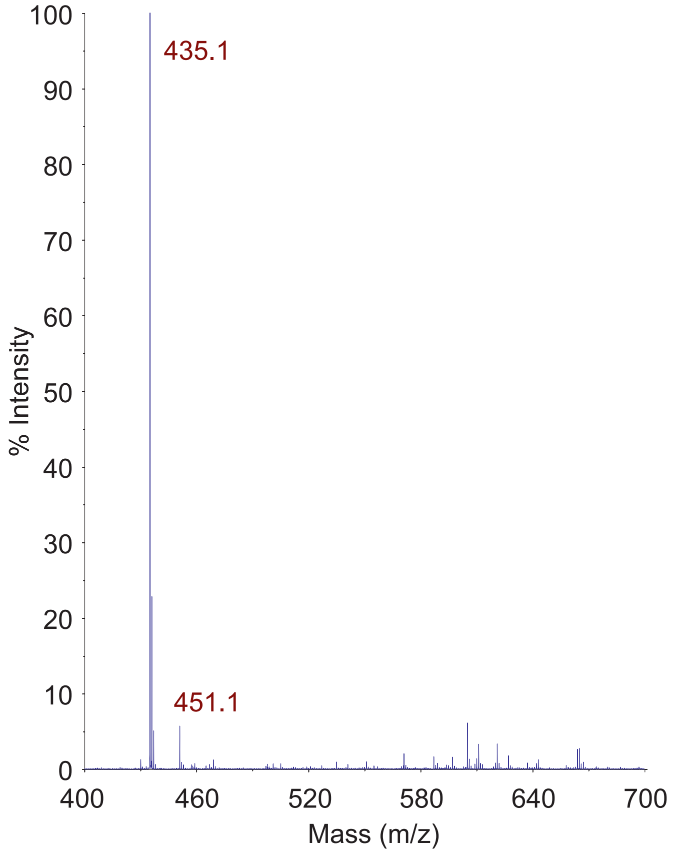

C Trehalose mono isobutyrate 435 (Na) / 451 (K)

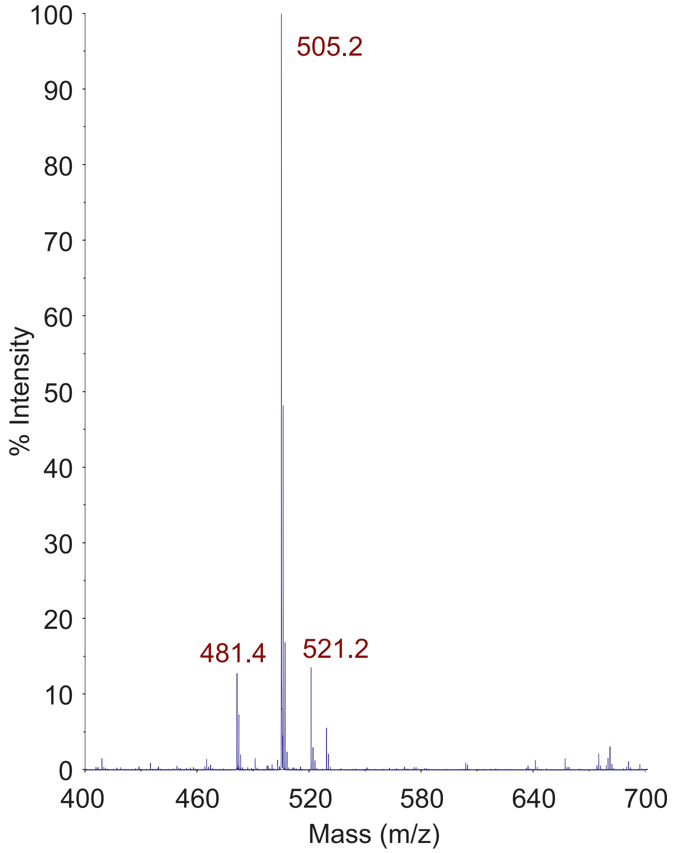

D Trehalose di isobutyrate 505 (Na) / 521(K)

Figure S2. Proton NMR spectra of acylated trehalose derivatives. Samples for NMR spectra, dissolved in D_2O at approximately 5 mg/ml, were analyzed on a Bruker 400 MHz spectrometer. Spectra are annotated to show signals from the acyl side chain (letters A-K) and key signals from trehalose. For monoacyl derivatives, signals from the non-acylated glucose residue are denoted with primes.

A Trehalose mono decanoate

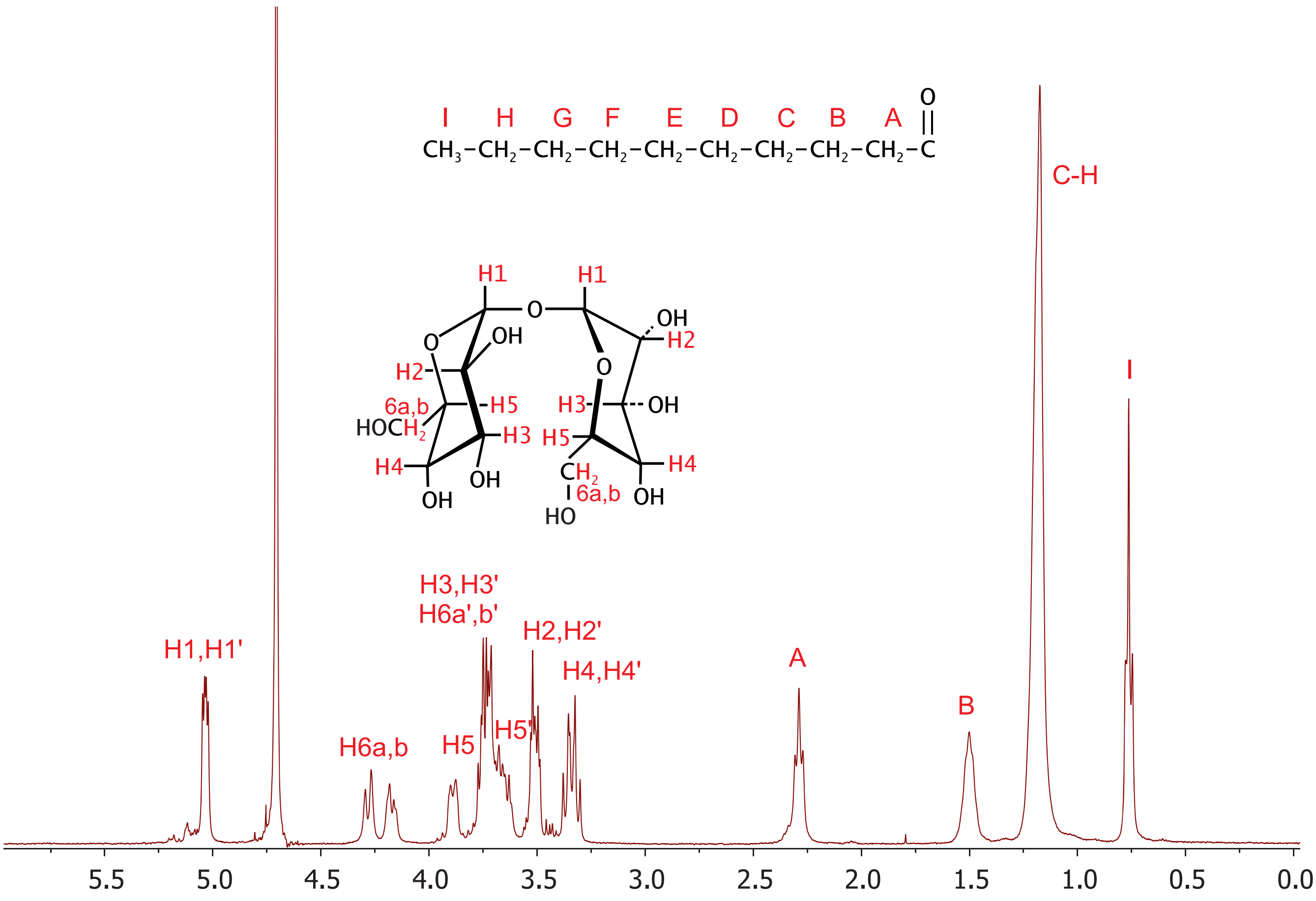

B Trehalose mono dodecanoate

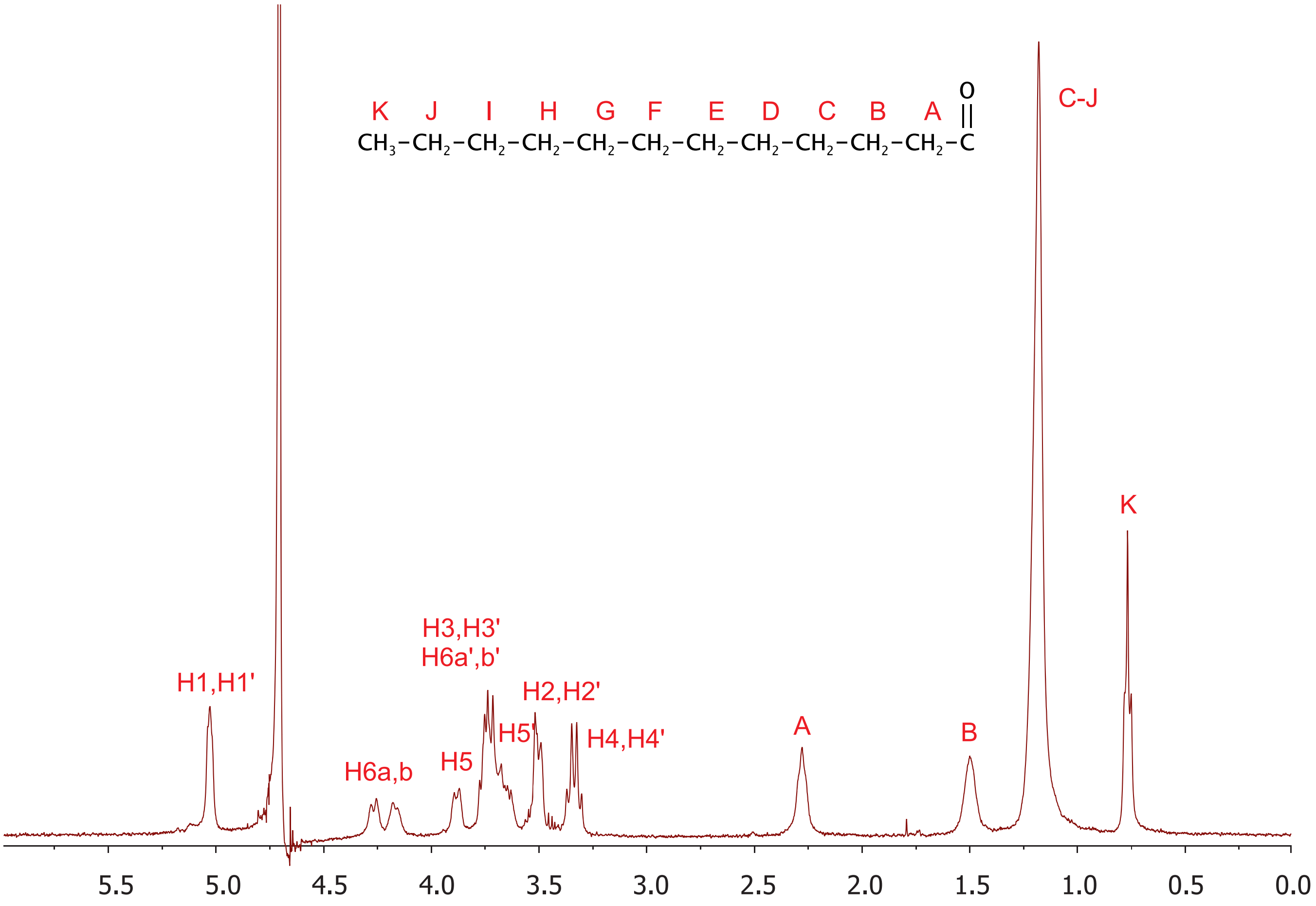

C Trehalose mono isobutyrate

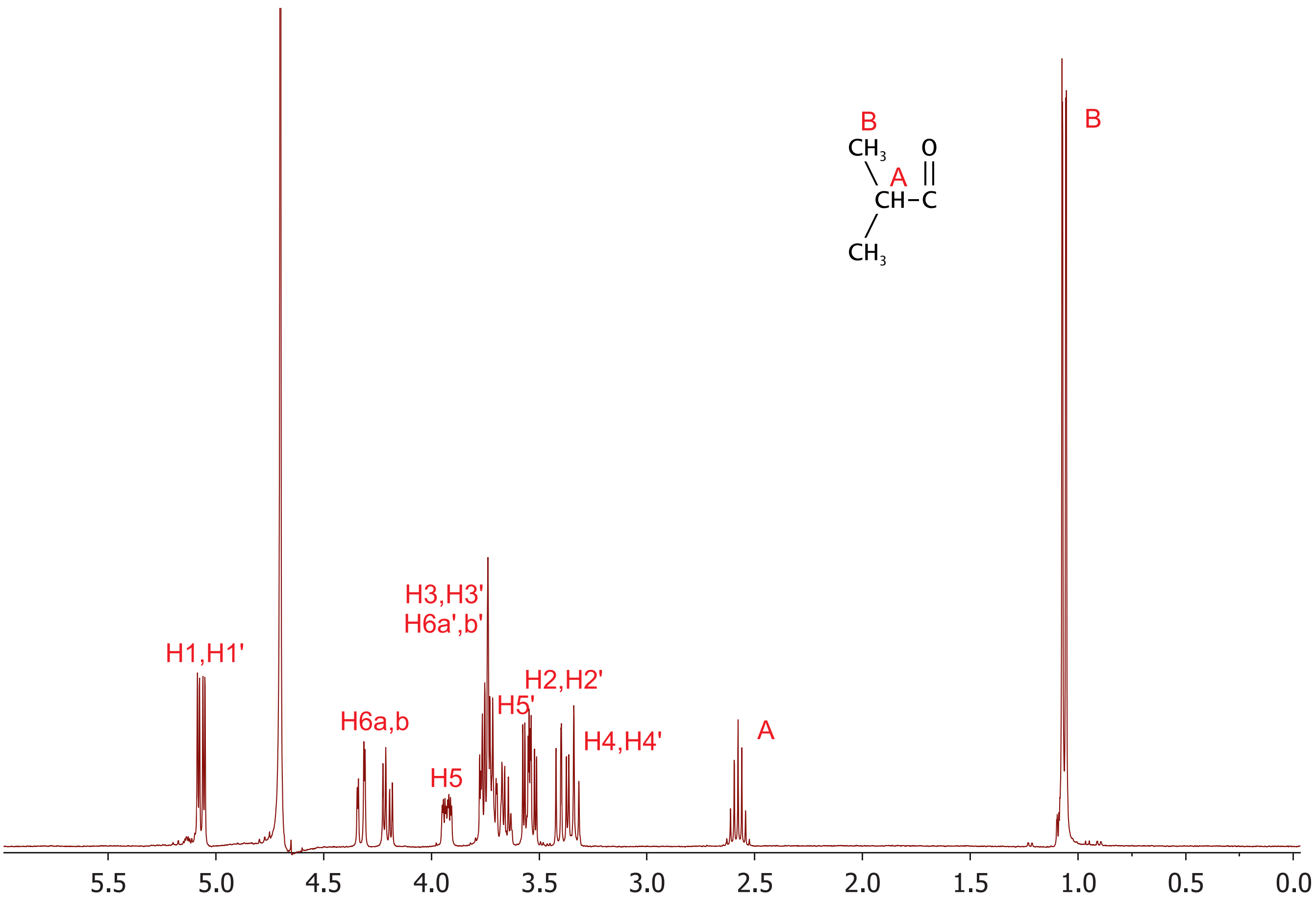

D Trehalose di isobutyrate

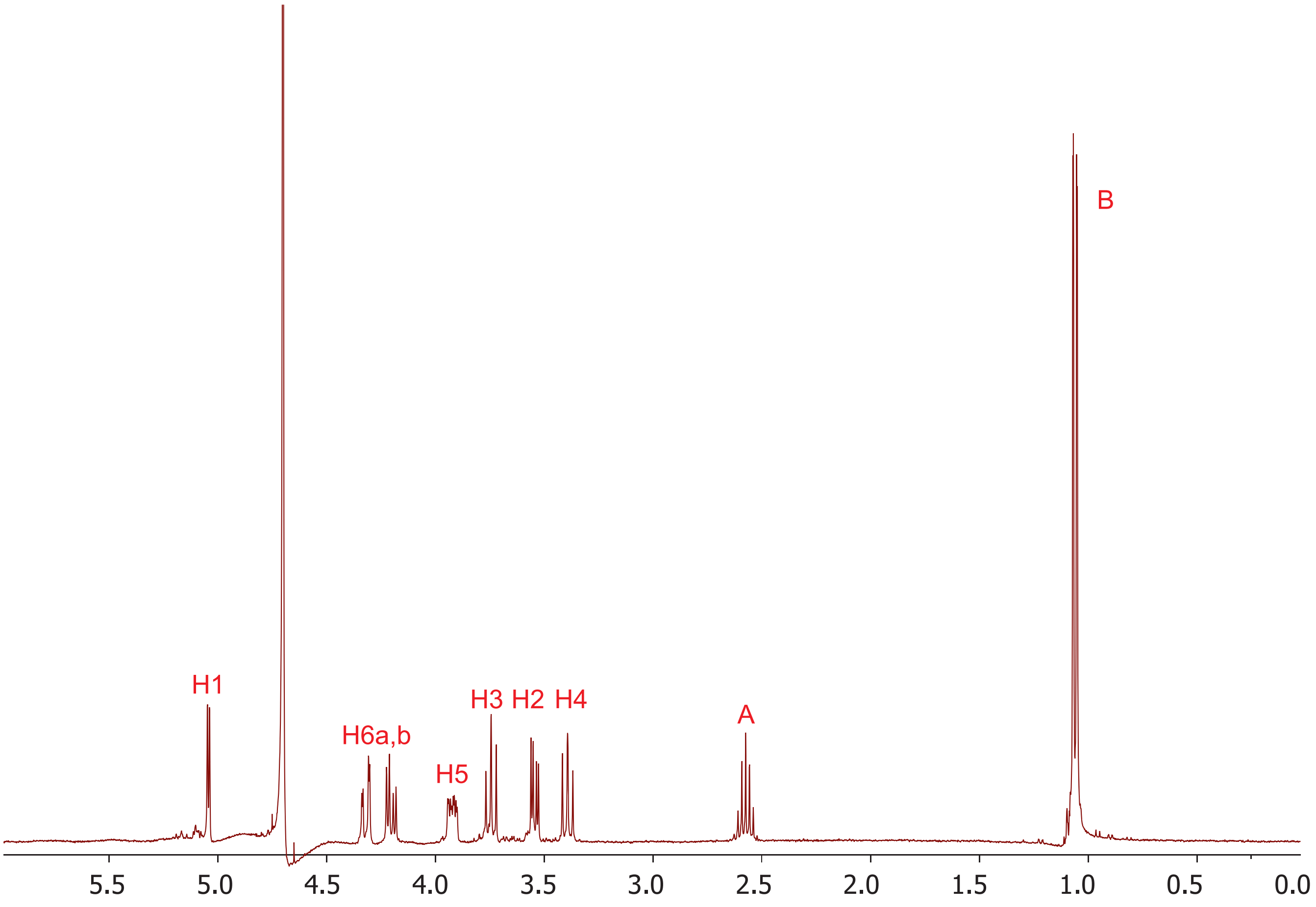

Supplement: Supplemental Data [file 10.1074_M116.749515_jbc.M116.749515-1.pdf]
